# Undernutrition in children under five associated with wealth-related inequality in 24 low- and middle-income countries from 2017 to 2022

**DOI:** 10.1038/s41598-024-53280-0

**Published:** 2024-02-09

**Authors:** Frehiwot Birhanu, Kiddus Yitbarek, Firew Tekle Bobo, Evan Atlantis, Mirkuzie Woldie

**Affiliations:** 1 School of Public Health, College of Health Science, Mizan-Tepi University, Mizan-Amana, Ethiopia; 2 School of Health Sciences, Western Sydney University, Penrith, NSW Australia; 3 Department of Health Policy and Management, Institute of Health, Jimma University, Jimma, Ethiopia; 4 School of Public Health, University of Technology Sydney, Sydney, NSW Australia; 5 Fenot Project, School of Population and Public Health, University of British Columbia, Addis Ababa, Ethiopia

**Keywords:** Health care, Medical research

## Abstract

Undernourishment is a persistent public health problem contributing to increased mortality in children under five in low-income countries, likely exacerbated by socio-economic disparities within communities. This paper aimed to examine the effect of wealth-related inequality on undernutrition in children under five in low, lower-middle, and upper-middle-income countries (LMICs). We analyzed cross-sectional data from the demographic and health survey program collected between 2017 and 2022 from 24 LMICs. Children born within 5 years preceding the survey were included in the analysis. Child undernutrition was the dependent variable (measured by stunting, wasting, and underweight) and country-level wealth-based inequality was the independent variable assessed by concentration index values stratified by the World Bank’s income categories. Within country inequality of child undernutrition was determined by concentration index (C) values with 95% confidence intervals (95% CI) and sub-group analysis by place of residence and sex of the child. We then fit bootstrapped meta-regression to check the variation in inequality of child undernutrition across different income category countries. The analysis was controlled by potential confounding variables. From the total sample size of 334,502 children included in the study, 35% were undernourished. Wealth-related inequality in child undernutrition was observed in 11 countries, consistently across income categories. Child undernutrition was highly concentrated among the poor households of Türkiye [C: − 0.26, 95% CI − 0.31 to − 0.20], and Cameroon [C: − 0.19, 95% CI − 0.22 to − 0.17], and relatively it was less concentrated among the poor in Liberia [C: − 0.07, 95% CI − 0.11 to − 0.04], and Gambia [C: − 0.07, 95% CI − 0.11 to − 0.04]. There is no difference in undernutrition associated with inequality between the three broad LMIC categories. The wealth-related inequality in child undernutrition within many of the included countries is still very significant. However, the economic category of countries made no difference in explaining wealth-related inequality in child undernutrition. Inter-sectoral collaboration to fight poverty and render special attention to the disadvantaged population segments would potentially help to address the observed inequity.

## Introduction

While global mortality in children under five has declined over recent decades, sub-national data for Low and Middle-Income Countries (LMICs) shows considerable raise especially in five sub-Sahara Africa (SSA)^1^. The relatively increased mortality in children under 5 years in LMICs is likely attributable to undernutrition, exacerbated by socio-economic disparities within communities^2,3^. Indeed, the United Nations recognizes that undernourishment and severe food insecurity have been increasing in most African countries^4^. Undernutrition in young children results in underweight, stunting, and wasting as an immediate outcome^5^. Strategies to address socio-economic disparities associated with undernutrition and subsequent mortality in young children are urgently needed^6^. 

The effect of childhood undernutrition is not limited to death and complications for the affected person. Undernutrition in early childhood has long-term consequences for the child and has intergenerational adverse effects^7,8^. Child undernutrition refers to an insufficient intake of energy and nutrients to meet the body’s need to maintain a state of health^9^. It is usually measured as either a combination of stunting (too short for age), underweight (too thin for age), wasting (too thin for height), or deficiencies in vitamins and minerals. Despite many targeted interventions globally, approximately 149 million (22.3%), 85 million (12.6%), and 45 million (6.6%) children under the age of five are estimated to have stunting, underweight, and wasting respectively in the world^10,11^.

There is substantial variation in the global distribution of undernutrition between different income countries. An aggregate data in 2020 showed the highest prevalence (50–55%) of childhood stunting in low-income countries like Timor, Burundi, and Eritrea, while it was as low as (1.5–2.0%) in high-income countries including Australia, Belgium, Chili, Greece, and Germany^12^. Similar disparities were observed for wasting with the highest prevalence in Djibouti (21%) and South Sudan (22%), whereas it was below 0.5% in Australia, Belgium, Chile, and the USA^13^. Furthermore, underweight was below 0.5% in Australia, Chile, Estonia, Germany, and the USA; and it was between 27 and 39% in Burundi, Chad, Djibouti, Sudan, and Eritrea^14^.

In addition to the disparities between countries of varying economic categories, socio-economic status is a common risk factor for child undernutrition within countries^15,16^. Evidence indicated that wealth and/or income of the household have a strong negative link with inequity in child undernutrition^17^. In countries such as Bangladesh, Pakistan, Nepal, India and Ethiopia the wealth-related gap in child undernutrition measures has been very high and increasing in some the areas^18–20^. On the other hand, the educational status of the mother, place of residence (rural/urban), and number of siblings are factors that create variation in the health status of the population including childhood level of nutrition^21^. Studies also uncover that seeking medical care whenever the child is ill^22^, and better child vaccination^23^ are protective factors against child undernutrition^24–27^.

Many economically better-off countries have now minimized and eliminated undernutrition^28,29^. Despite their success, the highest magnitude of undernutrition remains concentrated among the poorest economic group in many countries^28^. However, the extent to which the economically well-off countries managed the gap of undernutrition between the poorest and richest categories of the population remains unanswered. Therefore, this analysis set out to assess the wealth-based disparities in nutritional status among children under five in lower, lower-middle, and upper-middle-income countries. We further tested the effect of country level income category on equity differences in undernutrition.

## Methods

### Study design and setting

In this study, we used cross-sectional data collected from Low Income (LI), Lower Middle Income (LMI), and Upper-Middle-Income (UMI) countries by the Demographic and Health Survey (DHS) program. We categorized countries based on the 2020’s World Bank economic classification system^30^. A total of 11 LI countries that have a Gross National Income (GNI) per capita of 1035 US$ or less; eight LMI countries which have a GNI per capita between US$ 1036 and US$ 4045; and four UMI countries with a GNI per capita of between US$ 4046 and US$ 12,535 were included in the study. The population density of LI countries estimated to be 51.5 people per square kilometer and have a population growth rate of 2.6% per year^31^. Whereas the population growth rate for LMI countries is 1.3% per year and 135 people per square kilometer^31^. On the other side, the population density for UMI countries is 47 people per square kilometer, and the population growth rate is 0.2%^31^.

### Data sources and study participants

The information sources for this study were the DHS program of data collections from 24 countries that were published between 2017 and 2022. The included countries are from the three economic categories described earlier. Accordingly, Burundi (2016–17), Ethiopia (2019), Gambia (2019–20), Guinea (2018), Haiti (2017–18), Liberia (2019–20), Mali (2018), Niger (2017), Rwanda (2019–20), Sierra Leone (2019), and Tajikistan (2017) were from LI category; Bangladesh (2017–18), Benin (2017–18), Cameroon (2018), Mauritania (2019–21), Nigeria (2018), Pakistan (2017–18), Papua New Guinea (2016–18), and Senegal (2019) were LMI countries; and Albania (2017–18), Maldives (2016–17), India (2019–21), and Türkiye (2018) were UMI countries.

The DHS data was collected by dividing a country into enumeration areas (EA). An EA is a counting unit generated for the population census/survey. The DHS data being collected takes EAs as a sampling cluster, a further stage of sampling was then conducted to identify study participants. The DHS program collects data using five types of questionnaires. Among those, we considered children born in the 5 years before the interview, or the kids’ file component, and took children under the age of five as the study population. Questions related with the children’s module were answered by the mother/caregiver. We made use of the characteristics of the child, the mother/caregiver and the household in our analysis.

The DHS data are nationally representative data for population, family, and maternal and child health issues that provide data for a wide range of monitoring and impact evaluation indicators in the areas of population, health, and nutrition^32,33^. The DHS program works with country-specific agencies to conduct a household sample survey covering a range of population health indicators. The primary objective of the DHS is to provide up-to-date estimates of key demographic and health indicators^34^. The DHS datasets were obtained from the DHS Program website upon request^35^.

### Variables and measurement

#### Dependent variable

Undernutrition variables were estimated from measured body weight and height readings. Indicators of undernutrition were defined as stunting if the height-for-age Z-score is less than 2 standard deviations below the population mean; wasting if the weight for height Z-score is less than 2 standard deviations below the population mean, and similarly underweight if the weight-for-age Z-score is less than -2 standard deviations below the population mean^36^. We then classified undernutrition for the child participants with at least one of the above other indicators of undernutrition.

For a descriptive analysis purpose we calculated the proportion (prevalence) of all measures of undermatron (stunting, wasting and underweight) and overall undernutrition from overall children included in the analysis.

#### Independent variable

Regarding the household’s wealth, the DHS program uses the wealth index^37^ which serves as a composite score of the household’s living standard, considering the country’s context. The wealth index was calculated using easy-to-collect data on the household’s ownership of selected assets, and materials then, the final wealth quintile was obtained after a valuation and analysis using the principal component analysis tools^38^. The households were then categorized into either the poorest, poor, middle, rich, or richest quintile groups. To classify the region as urban or rural, the DHS program uses country-specific urban-to-rural classification methods and reports accordingly.

### Bias

The DHS data program collects data by dividing the country into different sizes of EAs and an equal amount of households taken as a sample from each of the EA. This end-up with a disproportionate sample size allocation to the EAs. We used sample and cluster weights to adjust for sampling errors.

### Statistical approach

We used concentration index (C) and concentration curves to assess the economic inequality in child nutritional status. Concentration curves were plotted considering the cumulative percentage of child undernutrition (y-axis) against the cumulative percentage of the population, ranked by wealth status variables (x-axis) (Supplementary file 1). To quantify equity differences, concentration index values were computed with the respective 95% confidence interval. The concentration index is twice the area between the concentration curve and the line of equality (the 45° line). The concentration index value ranges from − 1 to + 1. The convention is when the index value is negative the curve lies above the line of equality, indicating the disproportionate concentration of child undernutrition among the poor, and when it is positive it lies below the line of equality, showing the disproportionate child undernutrition among the rich^39^.

We did a sub-grouping analysis by place of residence and sex of the child. Additionally, a meta-regression test to check if there is a significant difference in nutritional status inequality among countries with different economic categories. For our regression model, we used the country-level wealth-based inequality in undernutrition as measured by concentration index values, and the countries’ economic category according to the World Bank’s definition was the independent variable^30^. We controlled the model with various variables including the country’s income inequality as measured by the Gini index^40^. Concentration index of undernutrition by maternal education: maternal education divided into four categories including no formal education, primary education, secondary education and higher education levels. Furthermore, we used concentration of undernutrition by number of under 5 years’ old children in the household, child vaccination status for Bacille Calmette-Guérin (BCG), one dose of pentavalent vaccine, and the first dose of measles-containing vaccine.

Considering the skewness of the distribution of the outcome variable and the few number of cases (24 countries), we employed the non-parametric bootstrapped regression with 1000 replications. We then tested the normal distribution of residuals. The variables with *p*-value < 0.05 was considered as significantly associated with the outcome variable. We used Stata version – 14 and Microsoft Excel spreadsheet version 2016 for the analysis. To display the level of child undernutrition in different countries and economic categories, the QGIS version 3.24.3 software was used. All the analyses were adjusted for clusters and sample weights.

We did aggregate level simple regression between level of undernutrition (stunting, wasting and underweight) as a dependent and the country’s overall income difference measure (Gini index value) as an independent variable. Because the distribution of the outcome variable is skewed to the right to all of the variables, we tend to use non-parametric non- bootstrapped regression with 1000 replications after checking log transformation is not working as well.

### Patient and public involvement

As the data sources were obtained from the DHS Program, there was no patient and public involvement.

### Ethical approval and informed consent

The authors analyzed publicly available secondary data obtained from the DHS program database. There was no additional ethical approval sought by the authors.

## Result

### Child nutritional status

**Figure 1:**
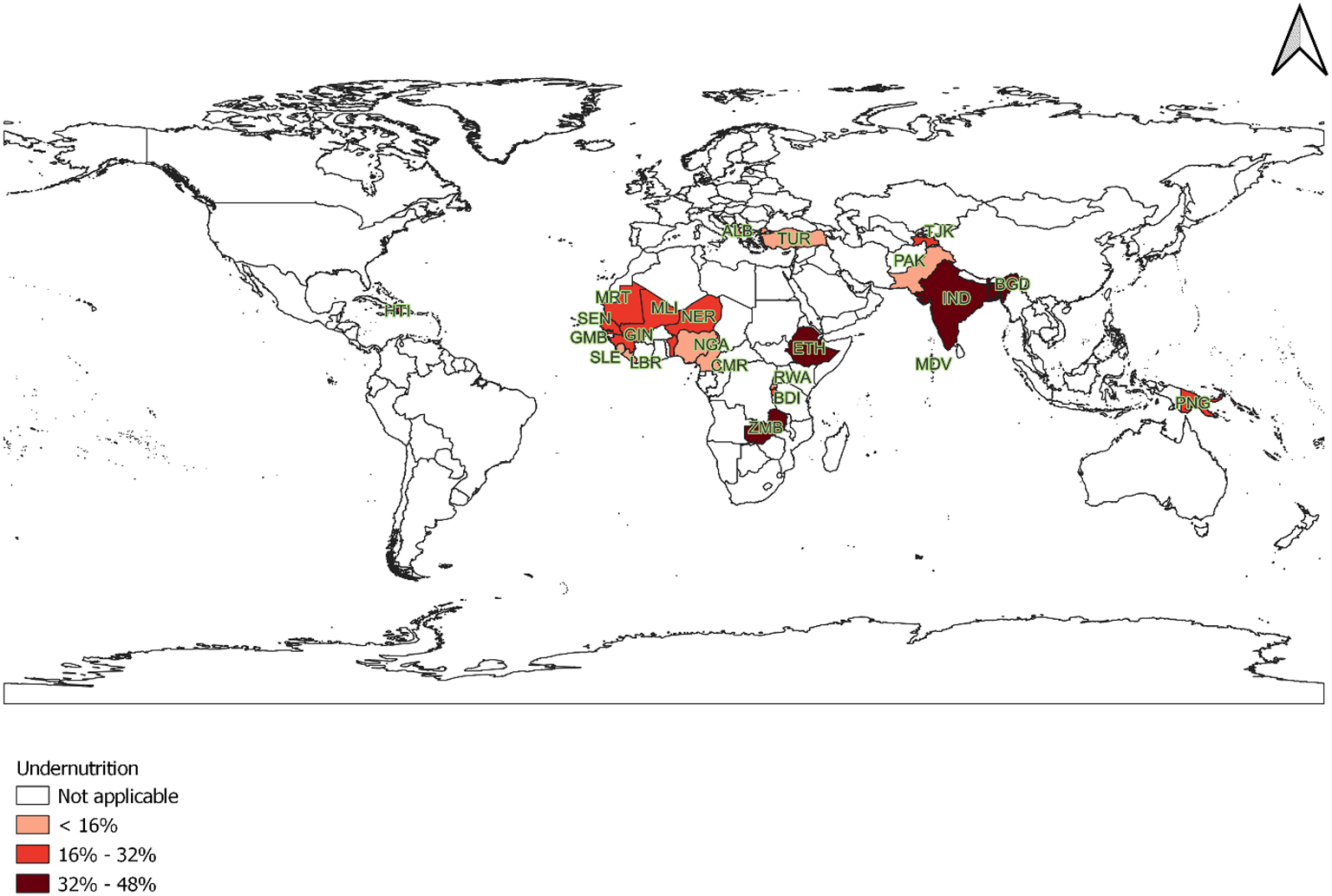
The status of child undernutrition in 24 lower, lower-middle, and upper-middle-income countries, 2017–2022.

Of the total sampled 24 countries, we obtained 334,502 for height-for-age, 334,071 for weight-for-height, and 331,541 for weight-for-age measures, respectively. There were 110,386 cases (33%) of stunting, 50,111 cases (15%) of wasting, and 89,516 cases (27%) of underweight yielding an overall child undernutrition rate of 35% (8.5% to 47.4%). The proportion of undernutrition was highest (47.4%) in India (UMIC), followed by Ethiopia, a LIC, (38.9%), and Bangladesh (35.1%) a LMIC. While it was lowest (8.5%) in Türkiye (UMIC), Gambia (LIC) (10.3%), and Albania (11.5%) which is a UMIC (Fig. 1) (The details are available in supplementary file 2).

### Wealth-related Inequalities in child undernutrition

Wealth-related inequality in child undernutrition status was observed in 12 out of 24 countries included in the analysis, consistently across all World Bank economic classifications (supplementary file 1 visually shows the wealth based variation in undernutrition). Furthermore, child undernutrition was highly concentrated among the poor households of Türkiye [C: − 0.26, 95% CI − 0.31 to − 0.20], Cameroon [C: − 0.19, 95% CI − 0.22 to − 0.17], and Haiti [C: − 0.18, 95% CI − 0.20 to − 0.15]. Undernutrition was relatively less concentrated among the poor in Liberia [C: − 0.07, 95% CI − 0.11 to − 0.04], and Gambia [C: − 0.07, 95% CI − 0.11 to − 0.04] (Table 1).

**Table 1:** Socio-economic inequalities defined using concentration indices in child undernutrition among 24 lower-, lower-middle, and upper-middle-income countries, 2017–2022.

Countries	Economic category	Stunting (95%CI)	Wasting (95%CI)	Underweight (95%CI)	Undernutrition (95%CI)
Overall		− 0.12 (− 0.13,− 0.12)*	− 0.08 (− 0.08,− 0.07)*	− 0.141 (− 0.144,− 0.138)*	− 0.096 (− 0.098,− 0.094)*
Albania	UMIC	− 0.17 (− 0.23,0.03)	− 0.08 (− 0.23,0.07)	− 0.1 (− 0.26,0.05)	− 0.17 (− 0.22,− 0.12)*
Bangladesh	LMIC	− 0.15 (− 0.17,− 0.13)*	− 0.06 (− 0.1,− 0.02)*	− 0.15 (− 0.17,− 0.12)*	− 0.13 (− 0.14,− 0.11) *
Benin	LIC	− 0.13 (− 0.29,− 0.11)*	0 (− 0.04,0.04)	− 0.12 (− 0.14,− 0.1)*	− 0.09 (− 0.1,− 0.08)*
Burundi	LIC	− 0.14 (− 0.15,− 0.12)*	− 0.18 (− 0.24,− 0.12)*	− 0.2 (− 0.22,− 0.18)*	− 0.12 (− 0.13,− 0.10) *
Cameroon	LMIC	− 0.19 (− 0.22,− 0.17)*	− 0.21 (− 0.29,− 0.12)*	− 0.33 (− 0.37,− 0.28)*	− 0.19 (− 0.22,− 0.17) *
Ethiopia	LIC	− 0.09 (− 0.11,− 0.07)*	− 0.24 (− 0.29,− 0.2)*	− 0.17 (− 0.2,− 0.14)*	− 0.10 (− 0.13,− 0.09*
Gambia	LIC	− 0.11 (− 0.15,− 0.07)*	0.02 (− 0.06,0.09)	− 0.09 (− 0.14, − 0.05)*	− 0.07 (− 0.11, − 0.04) *
Guinea	LIC	− 0.11 (− 0.13,− 0.08)*	− 0.02 (− 0.08,0.04)	− 0.11 (− 0.15, − 0.07)*	− 0.09 (− 0.12, − 0.06) *
Haiti	LIC	− 0.2 (− 0.23,− 0.17)*	0 (− 0.08,0.07)	− 0.19 (− 0.24, − 0.14)*	− 0.18 (− 0.20, − 0.15) *
India	UMIC	− 0.12 (− 0.124,− 0.118)*	− 0.07 (− 0.073,− 0.062)*	− 0.13 (− 0.137, − 0.13)*	− 0.09 (− 0.10,− 0.08) *
Liberia	LIC	− 0.07 (− 0.1,0.02)	0.12 (0.01,0.11)*	− 0.04 (− 0.1,0.43)	− 0.07 (− 0.11,− 0.04) *
Maldives	LMIC	− 0.06 (− 0.11,0.1)	− 0.02 (− 0.09,1.39)	− 0.07 (− 0.12,0.05)	− 0.06 (− 0.10,0.02)
Mali	LIC	− 0.15 (− 0.17,0.01)	− 0.08 (− 0.11,0.02)	− 0.14 (− 0.17,0.01)	− 0.11 (− 0.13,0.01)
Mauritania	LMIC	− 0.13 (− 0.15,0.01)	− 0.16 (− 0.2,0.02)	− 0.19 (− 0.21,0.01)	− 0.13 (− 0.15,0.01)
Niger	LIC	− 0.06 (− 0.08,0.01)	− 0.08 (− 0.12,0.02)	− 0.08 (− 0.11,0.01)	− 0.06 (− 0.08,0.01)
Nigeria	LMIC	− 0.22 (− 0.23,0.01)	− 0.18 (− 0.22,0.02)	− 0.24 (− 0.26,0.01)	− 0.12 (− 0.13,0.01)
Pakistan	LIC	− 0.18 (− 0.21,0.01)	− 0.14 (− 0.2,0.03)	− 0.26 (− 0.29,0.02)	− 0.16 (− 0.18,0.01)
Papua	LIC	− 0.14 (− 0.16,0.01)	− 0.03 (− 0.09,0.63)	− 0.12 (− 0.15,0.02)	− 0.09 (− 0.12,0.01)
Rwanda	LIC	− 0.21 (− 0.23,0.01)	− 0.11 (− 0.28,0.45)	− 0.26 (− 0.32,0.03)	− 0.20 (− 0.23,0.01)
Senegal	LMIC	− 0.18 (− 0.21,0.02)	− 0.09 (− 0.13,0.02)	− 0.18 (− 0.21,0.02)	− 0.14 (− 0.16,0.01)
Sierra Leone	LIC	− 0.09 (− 0.12,0.01)	0 (− 0.07,1.87)	− 0.05 (− 0.1,0.04)	− 0.08 (− 0.11,0.01)
Tajikistan	LIC	− 0.08 (− 0.11,0.02)	0.16 (0.1,0.03)	− 0.01 (− 0.06,1.34)	− 0.01 (− 0.04,0.61)
Türkiye	UMIC	− 0.27 (− 0.33,0.03)	− 0.05 (− 0.22,1.12)	− 0.23 (− 0.37,0.07)	− 0.26 (− 0.31,− 0.20)*
Zambia	LIC	− 0.08 (− 0.1,0.01)	0.01 (− 0.05,1.51)	− 0.11 (− 0.14,0.02)	− 0.07 (− 0.09,0.01)

*Significant at *p*-value < 0.05.

UMIC, upper middle-income country; LMIC, lower middle-income country; LIC, low-income country.

**Figure 2:**
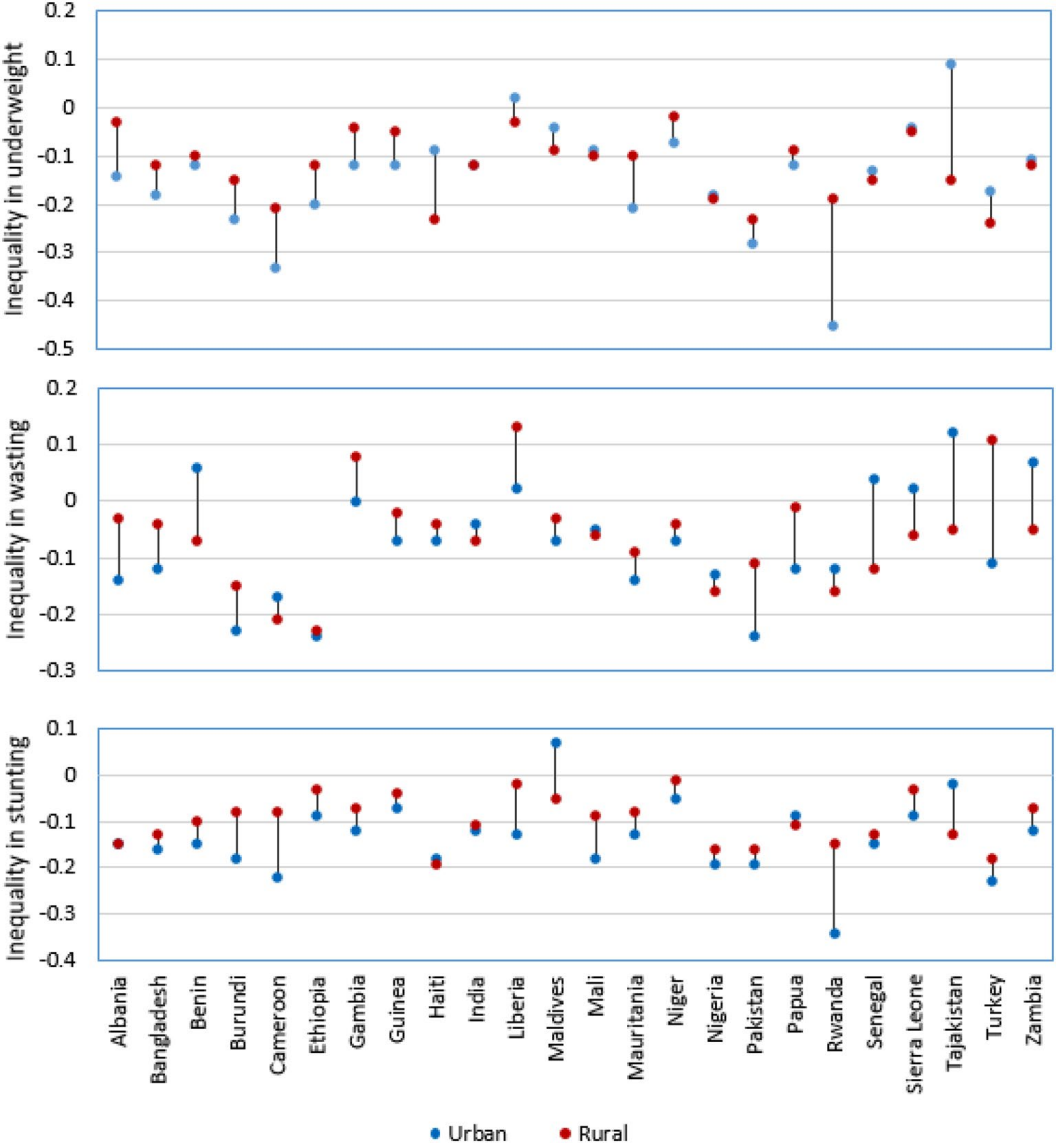
The status of urban to rural inequality gap in child undernutrition in the 24 lower, lower middle, and upper-middle-income countries.

The results indicated that there is more inequity in urban areas compared to the rural settings in most of the countries. For instance, if we see the urban–rural differences in stunting, the inequalities in Rwanda were on average 19% (C = − 0.19) more pronounced in the poor living in urban areas than in the rural. On the contrary, in Albania being poor in urban or rural communities did not show any difference. On the other side, the urban–rural equity gap in wasting was highest 22% (C = − 0.22) in poor communities of urban residents in Türkiye, whereas in Mali there was no inequality difference between urban and rural residents. Regarding underweight, the inequality difference was also higher (26% (C = − 0.26) in urban communities of Rwanda, and there was no inequality difference between urban and rural India. The overall gap in child undernutrition by place of residence was wider in Maldives (64%) and Rwanda (23%), whereas, in Sierra Leone, and Mali inequalities in child undernutrition were similar for urban and rural residents (Fig. 2) (Supplementary file 3).

Regarding inequalities within the sex of the child, the male-to-female difference in the concentration index of stunting was very close to zero in all of the countries. Coming to wasting, almost all countries had a minimal gap between male and female children, in Rwanda the gap was relatively higher C = 0.15. On the other side, inequalities for child underweight, and overall undernutrition was almost equal to zero for all countries. The same applies to overall undernutrition (Supplementary file 3).

### Factors affecting the wealth-related inequalities in child undernutrition

**Figure 3:**
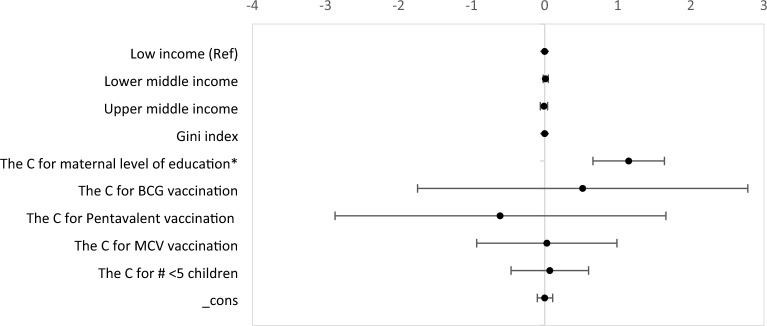
Factors affecting child undernutrition status in 24 selected low, lower middle, and upper-middle-income countries, 2017–2022.

We ran a bootstrapped regression using linear regression commands to test if there is any significant difference in the concentration index values because of the difference in the economic category of the country. The results of the test suggested that the concentration index of the countries did not significantly vary across different economic categories. The only variable that has a significant association with inequity in child undernutrition was the concentration of maternal level of education (Coef.: 1.15; 95% CI 0.66, 1.64) (Fig. 3) (details are available at supplementary file 4). Our sensitivity analysis has also revealed that there is no association between country level wealth variation and undernutrition measures (Supplementary file 5).

## Discussion

Our findings indicate, on average, one in three of the under-five child was undernourished regardless of the country’s economic status. This finding is consistent with other studies reporting that low-income countries are disproportionately affected by severe undernutrition^41,42^. Child undernutrition in these countries will undoubtedly cause severe health consequences for affected individuals throughout their lifespan^43^. Evidence shows that nutritionally disadvantaged children develop a range of health conditions including impaired cognitive function, resulting in further socio-economic disadvantage. This results in intergenerational social and economic disadvantage within low-income countries exacerbating the risk and adverse consequences of undernourishment in their vulnerable population groups^44,45^.

Apart from the differences in child undernutrition across the countries, wealth-related within-country inequality in childhood undernutrition was observed. In our study, a significant gap, which favors wealthier households, in all forms of child undernutrition was demonstrated. In countries like Burundi and Cameroon, the inequity was the highest. Earlier reports also indicated that wealth-related equity gaps in child undernutrition are common across all corners of the world^19,46^. This would be attributed to the economic status of the households which impacts their capability to ensure food security and to access health services whenever a child is ill^47,48^. Regardless of the success in minimizing the prevalence of undernutrition, countries are still challenged by an equity gap in child nutrition indicators. For instance, Cameroon has a 4.4% prevalence of stunting but has the highest gap among the poor and rich quintiles. Similarly, Haiti and Cameroon have the lowest prevalence of underweight. Meanwhile, the difference in underweight between children from the better off and the poorest family ranks among the countries with the highest inequity.

The other major concern related to child undernutrition status is the difference between being urban and rural residents and between sex groups. The concentration index values suggested that inequalities in child undernutrition were concentrated in poor communities of urban areas, than that of rural communities. In almost all countries, the three nutritional indicators have shown wider equity gaps in urban settings than the rural ones. This can be explained by the highly uncertain urban income which mainly comes from wage employment, trading, and self-employment which are more unstable and unequal than farming-based income sources^49,50^. In rural settings, life is relatively stable and relaxed, where people do agriculture and animal husbandry for a living creating a favorable environment to feed their children^51^. On the other hand, even though the gap is small, the equity gap in stunting is consistently higher among male children regardless of a country’s economic category^52^. Concerning wasting and being underweight in many countries, the equity gap is higher among female children. There is evidence indicating the prevalence of undernourishment is higher among female children in LMICs^53^.

Going through the analysis result for the 24 countries, one can observe significant inequity in child nutritional status regardless of a country’s economic category. Five (45.5%) of the countries from the low-income category, three (33.3%) countries from the lower middle-income category, and one (25%) country from the upper middle-income category of the countries have significant inequity in stunting of children favoring the highest wealth quintile. Consistently, our bootstrapped Meta-regression analysis revealed that there is no significant difference in the level of inequity of undernutrition in low, lower-middle, and upper-middle-income countries.

The overall finding implies that there is child undernutrition across the world and it gets worst in low-income countries. However, inequality among the poor and rich is prevalent in countries regardless of their economic category. The poor in low, lower-middle, and upper-middle-income countries are still disadvantaged regardless of the country’s economic status. When we relate this fact with the consequences of undernutrition, the existing situation forces the poor to live in cyclical poverty. If a child is undernourished, she/he will not be performing well in school and at work and consequently will earn less compared to those grown in a better condition^45,54,55^. Similarly, various chronic health problems and hospital admission among people who have a history of childhood undernourished are higher^7,56^. The intergenerational effect of undernutrition is also the other issue of concern. A child born from an undernourished mother is at a higher risk of death^8^, and would be less effective at the workplace^57^.

The strengths of this study include using nationally representative big sample data from 24 countries, and we did sub-group analysis by place of residence and sex of the child despite other studies focusing only on the wealth of the household. We also checked if there are differences in childhood undernutrition inequality in economically different countries descriptively and using a meta-regression analysis. The limitations include, as we have used DHS data, which is vulnerable to recall bias and may compromise the findings. Moreover, we used the most recent data from 24 countries of which some date back to 2016/17 which may not indicate the country’s current status in child undernutrition and wealth-related inequality.

## Conclusion

The findings of our analysis indicated that the magnitude of undernutrition varies across different income category countries, while inequity in all indicators of undernutrition is evident in all three economic groups. Therefore, stakeholders working to address the persistently high level of child undernourishment in low-income countries need to realize the double burden of high prevalence and deep inequality mainly affecting children of disadvantaged communities. Comprehensive interventions that target those the most vulnerable populations are urgently needed. Furthermore, better-off counties with a lesser burden of undernourishment should pay due attention to the unacceptably high equity gap that affects the less advantaged groups within affected countries. More importantly, fighting poverty and targeted treatment of the disadvantaged would potentially decrease undernutrition among the poor in countries of all economic categories.

### Supplementary Information

Supplementary Information.

## Data Availability

We used publicly available data from the Demographic and Health Survey database upon a reasonable request. Link to the DHS Program website: http://dhsprogram.com/data/available-datasets.cfm.
