# Supplementary material for: Undernutrition in children under five associated with wealth-related inequality in 24 low- and middle-income countries from 2017 to 2022
Source: Sci Rep. 2024 Feb 9;14:3326. doi: 10.1038/s41598-024-53280-0 (PMC10858243; doi:10.1038/s41598-024-53280-0)
Supplement: Supplementary file 1 — Supplementary Information. [file 41598_2024_53280_MOESM1_ESM.docx]

**Supplementary files**

**Supplementary file 1**

Figure: Concentration Curves that visualizes inequity in child undernutrition in each of 24 countries

**Albania Bangladesh**

**Benin Burundi**

**Cameroon Ethiopia**

**Gambia Guinea**

**Haiti India**

**Liberia Maldives**

**Mali Mauritania**

**Niger Nigeria**

**Pakistan Papua New Guinea**

**Rwanda Senegal**

**Sierra Leone Tajikistan**

**Turkey Zambia**

**Supplementary file 2**

**Table: Prevalence of undernutrition in low, lower-middle and upper middle countries, 2017-2022.**

| **Country** | **Region** | **Economic category** | **Stunting (%)** | **Wasting (%)** | **Underweight (%)** |
| --- | --- | --- | --- | --- | --- |
| Haiti | America | LI | 1132(21.2) | 204(3.8) | 487(9) |
| Tajikistan | Europe | LI | 1088(17.6) | 341(5.5) | 471(7.6) |
| Burundi | SSA | LI | 3472(55.8) | 318(5.1) | 1831(29.4) |
| Ethiopia | SSA | LI | 1833(36.8) | 364(7.2) | 1060(21.1) |
| Gambia | SSA | LI | 596(16.9) | 185(5.2) | 409(11.5) |
| Guinea | SSA | LI | 1048(31.1) | 302(9.1) | 553(16.1) |
| Liberia | SSA | LI | 624(28.8) | 81(3.7) | 224(10.3) |
| Mali | SSA | LI | 2367(26.7) | 800(8.9) | 1651(18.5) |
| Niger | SSA | LI | 2211(43.3) | 926(18.1) | 1849(36.2) |
| Rwanda | SSA | LI | 1294(33) | 45(1.2) | 294(7.5) |
| Sierra Leone | SSA | LI | 1177(29.1) | 226(5.6) | 557(13.6) |
| Pakistan | Eastern Mediterranean | LMI | 1299(37.3) | 243(7) | 812(22.7) |
| Bangladesh | South-east Asia | LMI | 2402(30.72) | 659(8.44) | 1753(21.8) |
| Benin | SSA | LMI | 3697(31.5) | 610(5) | 1958(16.6) |
| Cameroon | SSA | LMI | 206(4.4) | 1370(29.1) | 534(11.2) |
| Mauritania | SSA | LMI | 2530(25.4) | 613(6.2) | 1659(16.6) |
| Nigeria | SSA | LMI | 4165(36.5) | 793(6.9) | 2499(21.7) |
| Senegal | SSA | LMI | 899(17.6) | 409(8) | 720(14.1) |
| Zambia | SSA | LMI | 2976(34.6) | 367(4.3) | 1032(11.9) |
| Papua New Guinea | Western pacific | LMI | 305(9.2) | 1473(43.6) | 798(21.4) |
| Albania | Europe | UMI | 264(11.58) | 30(1.35) | 36(1.53) |
| Turkey | Europe | UMI | 240(9.5) | 44(1.7) | 49(1.9) |
| India | South-east Asia | UMI | 71566(35.5) | 38024(19.2) | 66118(32.1) |
| Maldives | South-east Asia | UMI | 306(15.1) | 184(9.2) | 319(15.2) |

**Supplementary file 3**

Table: Concentration index values of children undernutrition by type of place of residence (Urban-Rural) and sex of the child (Male-Female)

|  | Stunting |  |  |  |  |  | Wasting |  |  |  |  |  | Underweight |  |  |  |  |  |
| --- | --- | --- | --- | --- | --- | --- | --- | --- | --- | --- | --- | --- | --- | --- | --- | --- | --- | --- |
|  | Urban | Rural | U-R | Male | Female | M-F | Urban | Rural | U-R | Male | Female | M-F | Urban | Rural | U-R | Male | Female | M-F |
| Albania | -0.15(-0.25,-0.05) | -0.15(-0.22,-0.08) | 0 | -0.14(-0.22,-0.06) | -0.19(-0.28,-0.11) | 0.05 | -0.14(-0.38,0.1) | -0.03(-0.21,0.16) | -0.11 | -0.05(-0.34,0.13) | -0.06(-0.26,0.14) | 0.01 | -0.14(-0.38,0.1) | -0.03(-0.21,0.16) | -0.11 | -0.05(-0.34,0.13) | -0.06(-0.26,0.14) | 0.02 |
| Bangladesh | -0.16(-0.2,-0.13) | -0.13(-0.15,-0.1) | -0.03 | -0.15(-0.17,-0.12) | -0.16(-0.19,-0.13) | 0.01 | -0.12(-0.19,-0.05) | -0.04(-0.09,0.01) | -0.08 | -0.05(-0.1,0.01) | -0.07(-0.14,-0.01) | 0.02 | -0.18(-0.22,-0.14) | -0.12(-0.15,-0.09) | -0.06 | -0.15(-0.18,-0.11) | -0.15(-0.18,-0.11) | 0 |
| Benin | -0.15(-0.17,-0.13) | -0.1(-0.12,-0.08) | -0.05 | -0.13(-0.15,-0.11) | -0.14(-0.16,-0.12) | 0.01 | 0.06(0,0.12) | -0.07(-0.13,-0.01) | 0.13 | -0.05(-0.11,0.01) | 0.06(0,0.12) | -0.11 | -0.12(-0.16,-0.08) | -0.1(-0.12,-0.08) | -0.02 | -0.12(-0.16,-0.08) | -0.12(-0.16,-0.08) | 0 |
| Burundi | -0.18(-0.22,-0.14) | -0.08(-0.1,-0.07) | -0.1 | -0.13(-0.14,-0.11) | -0.15(-0.17,-0.13) | 0.02 | -0.23(-0.37,-0.08) | -0.15(-0.22,-0.08) | -0.08 | -0.19(-0.28,-0.11) | -0.17(-0.26,-0.07) | -0.02 | -0.23(-0.29,-0.17) | -0.15(-0.17,-0.13) | -0.08 | -0.19(-0.22,-0.16) | -0.21(-0.24,-0.18) | 0.02 |
| Cameroon | -0.22(-0.26,-0.17) | -0.08(-0.11,-0.05) | -0.14 | -0.18(-0.22,-0.15) | -0.2(-0.24,-0.16) | 0.02 | -0.17(-0.3,-0.04) | -0.21(-0.31,-0.11) | 0.04 | -0.22(-0.32,-0.11) | -0.19(-0.32,-0.06) | -0.03 | -0.33(-0.42,-0.24) | -0.21(-0.27,-0.16) | -0.12 | -0.3(-0.36,-0.24) | -0.36(-0.43,-0.28) | 0.06 |
| Ethiopia | -0.09(-0.13,-0.04) | -0.03(-0.05,-0.01) | -0.06 | -0.09(-0.12,-0.06) | -0.1(-0.13,-0.06) | 0.01 | -0.24(-0.33,-0.14) | -0.23(-0.28,-0.18) | -0.01 | -0.28(-0.34,-0.22) | -0.19(-0.26,-0.11) | -0.09 | -0.2(-0.25,-0.14) | -0.12(-0.15,-0.09) | -0.08 | -0.18(-0.21,-0.14) | -0.15(-0.2,-0.11) | -0.03 |
| Gambia | -0.12(-0.18,-0.06) | -0.07(-0.12,-0.03) | -0.05 | -0.1(-0.15,-0.05) | -0.13(-0.19,-0.07) | 0.03 | 0(-0.12,0.12) | 0.08(-0.02,0.17) | -0.08 | 0.05(-0.06,0.15) | -0.03(-0.15,0.1) | 0.08 | -0.12(-0.2,-0.05) | -0.04(-0.1,0.02) | -0.08 | -0.1(-0.16,-0.03) | -0.09(-0.16,-0.02) | -0.01 |
| Guinea | -0.07(-0.13,0) | -0.04(-0.07,-0.01) | -0.03 | -0.08(-0.12,-0.05) | -0.13(-0.18,-0.09) | 0.05 | -0.07(-0.18,0.04) | -0.02(-0.1,0.05) | -0.05 | -0.04(-0.13,0.04) | 0(-0.09,0.09) | -0.04 | -0.12(-0.21,-0.03) | -0.05(-0.09,0) | -0.07 | -0.12(-0.18,-0.06) | -0.1(-0.17,-0.04) | -0.02 |
| Haiti | -0.18(-0.24,-0.12) | -0.19(-0.22,-0.16) | 0.01 | -0.19(-0.23,-0.16) | -0.2(-0.25,-0.16) | 0.01 | -0.07(-0.2,0.06) | -0.04(-0.13,0.05) | -0.03 | -0.02(-0.12,0.08) | 0.02(-0.1,0.13) | -0.04 | -0.09(-0.18,0.01) | -0.23(-0.28,-0.17) | 0.14 | -0.22(-0.28,-0.15) | -0.16(-0.23,-0.08) | -0.06 |
| India | -0.12(-0.125,-0.108) | -0.11(-0.111,-0.104) | -0.01 | -0.12(-0.123,-0.114) | -0.12(-0.129,-0.12) | 0 | -0.04(-0.054,-0.03) | -0.07(-0.075,-0.064) | 0.03 | -0.07(-0.08,-0.066) | -0.06(-0.07,-0.055) | -0.01 | -0.12(-0.127,-0.108) | -0.12(-0.127,-0.119) | 0 | -0.13(-0.135,-0.125) | -0.14(-0.143,-0.133) | 0.01 |
| Liberia | -0.13(-0.2,0.03) | -0.02(-0.06,0.56) | -0.11 | -0.06(-0.1,0.04) | -0.08(-0.13,0.03) | 0.02 | 0.02(-0.16,1.79) | 0.13(0,0.16) | -0.11 | 0.1(-0.04,0.38) | 0.13(-0.03,0.27) | -0.03 | 0.02(-0.1,1.55) | -0.03(-0.1,0.9) | 0.05 | -0.06(-0.14,0.4) | -0.02(-0.11,1.38) | -0.04 |
| Maldives | 0.07(-0.13,1.05) | -0.05(-0.11,0.14) | 0.12 | -0.06(-0.13,0.28) | -0.05(-0.14,0.41) | -0.01 | -0.07(-0.3,1.25) | -0.03(-0.1,0.93) | -0.04 | -0.05(-0.14,0.63) | 0.04(-0.07,0.94) | -0.09 | -0.04(-0.22,1.32) | -0.09(-0.15,0.03) | 0.05 | -0.06(-0.14,0.26) | -0.08(-0.15,0.13) | 0.02 |
| Mali | -0.18(-0.23,0.02) | -0.09(-0.11,0.01) | -0.09 | -0.14(-0.17,0.01) | -0.16(-0.19,0.01) | 0.02 | -0.05(-0.12,0.29) | -0.06(-0.1,0.03) | 0.01 | -0.08(-0.13,0.03) | -0.07(-0.13,0.06) | -0.01 | -0.09(-0.15,0.03) | -0.1(-0.13,0.01) | 0.01 | -0.13(-0.16,0.02) | -0.16(-0.2,0.02) | 0.03 |
| Mauritania | -0.13(-0.16,0.02) | -0.08(-0.11,0.01) | -0.05 | -0.13(-0.15,0.01) | -0.14(-0.17,0.01) | 0.01 | -0.14(-0.22,0.04) | -0.09(-0.14,0.02) | -0.05 | -0.17(-0.23,0.03) | -0.15(-0.22,0.03) | -0.02 | -0.21(-0.25,0.02) | -0.1(-0.13,0.01) | -0.11 | -0.18(-0.21,0.02) | -0.2(-0.23,0.02) | 0.02 |
| Niger | -0.05(-0.08,0.02) | -0.01(-0.03,0.72) | -0.04 | -0.06(-0.09,0.01) | -0.06(-0.09,0.01) | 0 | -0.07(-0.12,0.06) | -0.04(-0.08,0.05) | -0.03 | -0.06(-0.11,0.03) | -0.11(-0.16,0.03) | 0.05 | -0.07(-0.11,0.02) | -0.02(-0.04,0.21) | -0.05 | -0.08(-0.11,0.02) | -0.09(-0.12,0.02) | 0.01 |
| Nigeria | -0.19(-0.22,0.01) | -0.16(-0.18,0.01) | -0.03 | -0.21(-0.23,0.01) | -0.22(-0.24,0.01) | 0.01 | -0.13(-0.2,0.04) | -0.16(-0.2,0.02) | 0.03 | -0.17(-0.22,0.03) | -0.2(-0.27,0.03) | 0.03 | -0.18(-0.22,0.02) | -0.19(-0.22,0.01) | 0.01 | -0.23(-0.26,0.01) | -0.25(-0.28,0.01) | 0.02 |
| Pakistan | -0.19(-0.22,0.02) | -0.16(-0.19,0.01) | -0.03 | -0.18(-0.21,0.01) | -0.19(-0.22,0.02) | 0.01 | -0.24(-0.32,0.04) | -0.11(-0.19,0.05) | -0.13 | -0.13(-0.21,0.04) | -0.16(-0.24,0.04) | 0.03 | -0.28(-0.33,0.03) | -0.23(-0.27,0.02) | -0.05 | -0.25(-0.3,0.02) | -0.26(-0.31,0.02) | 0.01 |
| Papua | -0.09(-0.14,0.03) | -0.11(-0.14,0.01) | 0.02 | -0.11(-0.14,0.02) | -0.17(-0.2,0.02) | 0.06 | -0.12(-0.22,0.1) | -0.01(-0.08,1.68) | -0.11 | -0.02(-0.11,1.24) | -0.04(-0.13,0.73) | 0.02 | -0.12(-0.19,0.04) | -0.09(-0.12,0.02) | -0.03 | -0.11(-0.15,0.02) | -0.13(-0.18,0.03) | 0.02 |
| Rwanda | -0.34(-0.41,0.04) | -0.15(-0.18,0.01) | -0.19 | -0.2(-0.23,0.02) | -0.22(-0.26,0.02) | 0.02 | -0.12(-0.43,1.08) | -0.16(-0.35,0.28) | 0.04 | -0.03(-0.28,1.8) | -0.18(-0.4,0.33) | 0.15 | -0.45(-0.64,0.09) | -0.19(-0.26,0.03) | -0.26 | -0.22(-0.3,0.04) | -0.32(-0.42,0.05) | 0.1 |
| Senegal | -0.15(-0.22,0.03) | -0.13(-0.17,0.02) | -0.02 | -0.14(-0.18,0.02) | -0.22(-0.27,0.02) | 0.08 | 0.04(-0.05,0.86) | -0.12(-0.17,0.03) | 0.16 | -0.11(-0.17,0.03) | -0.06(-0.13,0.29) | -0.05 | -0.13(-0.21,0.04) | -0.15(-0.19,0.02) | 0.02 | -0.15(-0.19,0.02) | -0.22(-0.27,0.02) | 0.07 |
| Sierra Leone | -0.09(-0.15,0.03) | -0.03(-0.06,0.05) | -0.06 | -0.07(-0.1,0.02) | -0.11(-0.15,0.02) | 0.04 | 0.02(-0.1,1.63) | -0.06(-0.15,0.32) | 0.08 | 0.01(-0.08,1.58) | -0.01(-0.12,1.81) | 0.02 | -0.04(-0.11,0.76) | -0.05(-0.1,0.11) | 0.01 | -0.04(-0.1,0.4) | -0.07(-0.14,0.08) | 0.03 |
| Tajakistan | -0.02(-0.06,0.88) | -0.13(-0.17,0.02) | 0.11 | -0.07(-0.11,0.02) | -0.09(-0.13,0.02) | 0.02 | 0.12(0.06,0.03) | -0.05(-0.13,0.41) | 0.17 | 0.17(0.1,0.04) | 0.14(0.06,0.04) | 0.03 | 0.09(0.03,0.04) | -0.15(-0.21,0.03) | 0.24 | 0(-0.06,1.9) | -0.03(-0.1,0.89) | 0.03 |
| Turkey | -0.23(-0.32,0.04) | -0.18(-0.26,0.04) | -0.05 | -0.25(-0.34,0.04) | -0.29(-0.39,0.05) | 0.04 | -0.11(-0.32,0.69) | 0.11(-0.13,0.87) | -0.22 | -0.05(-0.3,1.51) | -0.06(-0.28,1.32) | 0.01 | -0.17(-0.35,0.21) | -0.24(-0.43,0.12) | 0.07 | -0.25(-0.45,0.12) | -0.21(-0.4,0.16) | -0.04 |
| Zambia | -0.12(-0.15,0.02) | -0.07(-0.08,0.01) | -0.05 | -0.07(-0.1,0.01) | -0.09(-0.11,0.01) | 0.02 | 0.07(-0.03,0.35) | -0.05(-0.12,0.33) | 0.12 | 0(-0.08,1.89) | 0.02(-0.06,1.17) | -0.02 | -0.11(-0.17,0.03) | -0.12(-0.15,0.02) | 0.01 | -0.1(-0.15,0.02) | -0.12(-0.16,0.02) | 0.02 |

**Supplementary file 4**

Table: Factors affecting child undernutrition status in 24 selected low, lower middle, and upper-middle-income countries, 2017-2022.

| **Variables** | **Simple regression model** | |  | **Multiple regression model** | | |
| --- | --- | --- | --- | --- | --- | --- |
|  | **Coef. (95% CI)** | **P-value** |  | **Coef. (95% CI)** | **Std. Err.** | **P-value** |
| Economic category |  |  |  |  |  |  |
| LI |  |  |  | Reference |  |  |
| LMI | -0.03(-0.08,0.02) | 0.3 |  | 0.01 (-0.02, 0.05) | 0.02 | 0.47 |
| UMI | -0.04(-0.11,0.02) | 0.19 |  | -0.01 (-0.06, 0.04) | 0.02 | 0.66 |
| Gini index | -0.002(-0.006,0.002) |  |  | 0.00 (0.00, 0.00) | 0.00 | 0.59 |
| The C for maternal level of education | 1.04(0.83,1.25) | <0.01 |  | 1.15 (0.66, 1.64)* | 0.25 | <0.01* |
| The C for BCG vaccination | 1.51(0.57, 2.46) | <0.01 |  | 0.52 (-1.74, 2.78) | 1.15 | 0.65 |
| The C for Pentavalent vaccination | 1.13(0.43, 1.84) | <0.01 |  | -0.61 (-2.87, 1.66) | 1.16 | 0.60 |
| The C for MCV vaccination | 0.46(-0.21, 1.14) | 0.17 |  | 0.03 (-0.93, 0.99) | 0.49 | 0.95 |
| The C for # <5 children | -0.47(-1.09,0.15) | 0.13 |  | 0.07 (-0.46, 0.60) | 0.27 | 0.80 |
| _cons |  |  |  | 0.00 (-0.10, 0.11) | 0.05 | 0.96 |

**Supplementary file 5**

**Sensitivity analysis**

Table: Sensitivity analysis using stunting and country level Gini Index

|  |  |  | Number of obs | = | 24 |
| --- | --- | --- | --- | --- | --- |
|  |  |  | Replications | = | 1000 |
|  |  |  | Wald chi2(1 | = | 0.14 |
|  |  |  | Prob > chi2 | = | 0.713 |
|  |  |  | R-squared | = | 0.0084 |
|  |  |  | Adj R-squared | = | -0.0367 |
|  |  |  | Root MSE | = | 12.4467 |
|  |  |  |  |  |  |
|  | Coef. | Bootstrap Std. Err. | p-value | Normal-based 95% CI | |
|  |  |  |  | Lower | Upper |
| Gini Index | -0.18 | 0.49 | 0.71 | -1.14 | 0.78 |
| _cons | 33.42 | 17.77 | 0.06 | -1.39 | 68.24 |

Table: Sensitivity analysis using wasting and country level Gini Index

|  |  |  | Number of obs | = | 24 |
| --- | --- | --- | --- | --- | --- |
|  |  |  | Replications | = | 1000 |
|  |  |  | Wald chi2(1 | = | 0.15 |
|  |  |  | Prob > chi2 | = | 0.6955 |
|  |  |  | R-squared | = | 0.0121 |
|  |  |  | Adj R-squared | = | -0.0329 |
|  |  |  | Root MSE | = | 9.7708 |
|  |  |  |  |  |  |
|  | Coef. | Bootstrap Std. Err. | p-value | Normal-based 95% CI | |
|  |  |  |  | Lower | Upper |
| Gini Index | 0.17 | 0.43 | 0.70 | -0.68 | 1.02 |
| _cons | 2.91 | 14.83 | 0.85 | -26.16 | 31.97 |

Table: Sensitivity analysis using Underweight and country level Gini Index

|  |  |  | Number of obs | = | 24 |
| --- | --- | --- | --- | --- | --- |
|  |  |  | Replications | = | 1000 |
|  |  |  | Wald chi2(1 | = | 1.9 |
|  |  |  | Prob > chi2 | = | 0.1683 |
|  |  |  | R-squared | = | 0.0622 |
|  |  |  | Adj R-squared | = | 0.0196 |
|  |  |  | Root MSE | = | 8.5658 |
|  |  |  |  |  |  |
|  | Coef. | Bootstrap Std. Err. | p-value | Normal-based 95% CI | |
|  |  |  |  | Lower | Upper |
| Gini Index | -0.35 | 0.25 | 0.17 | -0.84 | 0.15 |
| _cons | 29.32 | 9.76 | 0.00 | 10.19 | 48.44 |
